# Uterine and placental expression of TRPV6 gene is regulated via progesterone receptor- or estrogen receptor-mediated pathways during pregnancy in rodents

**DOI:** 10.1186/1477-7827-7-49

**Published:** 2009-05-21

**Authors:** Bo-Mi Lee, Geun-Shik Lee, Eui-Man Jung, Kyung-Chul Choi, Eui-Bae Jeung

**Affiliations:** 1 Laboratory of Veterinary Biochemistry and Molecular Biology, College of Veterinary Medicine, Chungbuk National University, Cheongju, Chungbuk, 361-763, Republic of Korea

## Abstract

Transient receptor potential cation channel, subfamily V, member 6 (TRPV6) is an epithelial Ca^2+ ^channel protein expressed in calcium absorbing organs. In the present study, we investigated the expression and regulation of uterine and placental TRPV6 during gestation in rodents. Uterine TRPV6 peaked at pregnancy day (P) 0.5, P5.5 and, P13.5 and was detected in uterine epithelium and glands of rats, while placental TRPV6 mRNA levels increased in mid-gestation. Uterine and placental TRPV6 mRNA levels in rats appear to cyclically change during pregnancy, suggesting that TRPV6 may participate in the implantation process. In addition, uterine TRPV6 mRNA is only expressed in placenta-unattached areas of the uterus, and uterine TRPV6 immunoreactivity was observed in luminal and glandular epithelial cells. In the placenta, TRPV6 was detected in the labyrinth and spongy zone. These results may indicate that TRPV6 has at least two functions: implantation of the embryo and maintenance of pregnancy. To investigate the pathway(s) mediating TRPV6 expression in rodents, anti-steroid hormone antagonists were injected prior to maximal TRPV6 expression. In rats, TRPV6 expression was reduced by RU486 (an anti-progesterone) through progesterone receptors, and ICI 182,780 (an anti-estrogen) blocked TRPV6 expression via estrogen receptors in mice. The juxtaposition of uterine and placental TRPV6 expressed in these tissues supports the notion that TRPV6 participates in transferring calcium ions between the maternal and fetal compartments. Taken together, TRPV6 gene may function as a key element in controlling calcium transport in the uterus between the embryo and the placenta during pregnancy.

## Background

Uterine calcium ions are considered to be a critical factor for smooth muscle contraction and embryo implantation. At the implantation stage, these ions may help in secreting uterine extra-cellular matrix components at the site of implantation, and during pregnancy and labor calcium ions may regulate uterine tension [1,2]. Despite these critical roles of calcium, the regulation of uterine calcium levels is not yet fully understood. In calcium absorbing organs, i.e., intestine and kidney which express several calcium processing proteins, the regulation and function of calcium ions and the associated genes are relatively well understood. Calcium ions are processed via entering, transporting, and extruding proteins [3-5]. The transient receptor potential cation channel, subfamily V, member (TRPV) 5 and 6 were found in apical membranes of intestinal and renal epithelial cells, and are proposed as mediators for calcium uptake during trans-cellular transport [6]. Cytosolic calbindin-D9k and -28 k transport calcium ions from apical to basolateral layers, and theses ions are finally extruded from the cell membrane via plasma membrane Ca^2+ ^ATPase and sodium-calcium exchanger 1 [4,5]. In the past decade, uterine calcium-binding proteins have been studied to elucidate the function of uterine calcium ions, however their regulation and mechanisms of action are not completely resolved [7-9].

In female reproductive organs, *TRPV6 *is expressed in the uterus as well as in the placenta [10-12]. In calcium absorbing organs, TRPV6 expression is regulated by vitamin D, estrogen and dietary calcium. An active form of vitamin D increases duodenal calcium absorption, and abnormally low calcium absorption has been observed in vitamin D receptor-knockout mice [4,13]. Dietary calcium can also induce duodenal and renal *TRPV6 *mRNA expression [5,14], and estrogen therapy in menopausal women induces duodenal *TRPV6 *mRNA, suggesting that this hormone independently modulates TRPV6 expression [15]. TRPV6 of the female reproductive organs is robustly expressed; however its exact role is reproduction is not clearly understood. In the previous study, we reported that rat *TRPV6 *is expressed in the uterine endometrium and glandular endometrium [11,12] and is up-regulated at diestrus in matured animals and by progesterone supplementation in immature or ovariectomized animals [11]. Recently, the physiological significance of TRPV6/Ca^2+ ^channel in maternal-fetal Ca^2+ ^transport was investigated using TRPV6 knockout mice [16], demonstrating that Ca^2+ ^concentration in fetal blood and amniotic fluid was significantly lower and the transport activity of radioactive Ca^2+ ^from mother to fetuses was 40% lower in TRPV6 KO fetuses than in WTs. Progesterone appears to be a dominant regulator of *TRPV6 *via progesterone receptor activation [11]. In a mouse model, uterine *TRPV6 *expression varied during estrous cycles, however it was dominantly observed at the estrous stage [12]. In addition, estrogen seemed to be a major factor in the regulation of uterine *TRPV6 *transcription via estrogen receptor α pathway [12]. However, the role of TRPV6 in the implantation process during pregnancy is not yet determined.

Thus, in the present study, we employed a rat model to examine the expression of *TRPV6 *mRNA in the uterus and placenta during pregnancy. In addition, we investigated whether sex hormones regulate uterine *TRPV6 *expression in rats and mice during pregnancy, using antagonists for estrogen and progesterone. Finally, we further determined the localization of TRPV6 protein in the uterus and placenta to elucidate the role of this protein during pregnancy.

## Methods

### Animals and treatments

Sprague-Dawley rats (male, 12-week-old; female, 10-week-old) and pregnant ICR mice (12 to 15-week-old) were obtained from KOATECH (Pyeongtaek, Gyeonggi, Korea). All animals were housed in polycarbonate cages, and acclimatized to an environmentally controlled room before experimentation (temperature, 23 ± 2°C; relative humidity, 50 ± 10%; frequent ventilation and 12 h light cycle). Experiments were performed with the approval of the Animal Ethics Committee at the College of Veterinary Medicine, Chungbuk National University. Adult female rats were mated with adult males overnight [17], then examined the following morning for the presence of a vaginal plug. This time point was designated as day 0.5 of pregnancy (P0.5) when the morning a plug is found. The rats (n = 3 per group) were euthanized on each day of pregnancy (P0.5 to P21.5).

Pregnant rats (P4.5 for uterine RNA preparation, P19.5 for placental RNA preparation) received subcutaneous injections of RU486 (RU, 2.5 mg per rat; Sigma-Aldrich Corp, St. Louis, MO), ICI 182,780 (ICI, 0.5 mg per rat; Tocris, Eslisvill, Missouri, USA) or 50% ethanol as a vehicle [11,18]. In addition, ICR mice at P9.5 were injected with RU (25 μg per mouse) or ICI (2 μg per mouse) [19]. The chemicals were dissolved in 0.2 ml ethanol and saline mixture (1:1), and 0.2 ml and 0.05 ml were administered to rats and mice, respectively. The pregnant rats were treated with the antagonists one day before *TRPV6 *mRNA is maximally expressed depending on the tissues and species [12].

### Total RNA extraction and reverse transcriptase (RT)-PCR

Animals were euthanized, and the uteri and placentas were rapidly excised and washed in cold sterile saline (0.9% NaCl). Total RNA was prepared with TRIzol reagent (Invitrogen Life Technologies, Inc., Carlsbad, California, USA), and the concentration was determined by measuring light absorbance at 260 nm. Before RT-PCR, total RNA samples were electrophoresed on 1% formaldehyde denaturing agarose gels to validate the quality and purity. RT-PCR was performed, and the resulting products were visualized by agarose gel electrophoresis as described previously [20]. In brief, total RNA (1 μg) was reverse transcribed to first strand complementary DNA (cDNA) using mMLV reverse transcriptase (Invitrogen Life Technologies, Inc.) and random primers (9 mers; TaKaRa Bio. Inc., Otsu, Shiga, Japan). *TRPV6 *and *1A *(cytochrome oxidase subunit I, a house keeping gene) were amplified in a 20 μl PCR reaction containing 1 U Taq polymerase (iNtRON Bio Inc, Sungnam, Kyungki-Do, Korea), 1.5 mM MgCl_2_, 2 mM dNTP, and 50 pmol *TRPV6*- or *1A *specific primers [12]. The oligonucleotide sequences for *TRPV6 *were 5'-GTG CTG GGT GCC ATC TAC GT-3' (sense) and 5'-CAA TGA TGA CAT GGA ATG GCC-3' (antisense). The primer sequences for *1A *were 5'-CCA GGG TTT GGA ATT ATT TC-3' (sense) and 5'-GAA GAT AAA CCC TAA GGC TC-3' (antisense). PCR reactions were denatured at 95°C for 30 s, annealed at 60°C for 30 s and extended at 72°C for 45 s. *TRPV6 *and *1A *were quantified after 25 and 18 cycles, respectively. PCR products (10 μl) were separated on a 2% agarose gel, stained with ethidium bromide, and photographed under UV illumination. Photographs were taken using a Gel Doc EQ (Bio-Rad, Hercules, California, USA).

### Real-time PCR using TaqMan™ probe

Real-time PCR was performed in 20 μl reactions containing 10 μl TaqMan Universal PCR Master Mix (Applied Biosystems, Foster, CA, USA), and 1 μl of 20×Assays-on-Demand ™ Gene Expression Assay Mix (Applied Biosystems; rat *TRPV6*, Rn00586673_A1 [Assay ID in the Applied Biosystmes]; mouse *TRPV6*, Mm00499069_m1; rat *HPRT1 *(hypoxanthine guanine phosphoribosyl transferase 1, an internal control), Rn01527840_A1; mouse *HPRT1*, Mm00446968_m1) and 2 μl cDNA. PCR amplification was conducted using a 7300 Real-Time PCR System (Applied Biosystems), with initial enzyme activation at 50°C for 2 min, followed by 90°C for 10 min. Each of 40 amplification cycles consisted of denaturation at 95°C for 15 sec, followed by annealing and extension at 60°C for 1 min. Relative expression levels for each sample were determined using RQ software (Applied Biosystems). The expression of *TRPV6 *was normalized relative to that of *HPRT1 *[21,22]

### Immunohistochemistry

The tissue localization of TRPV6 protein was examined by immunohistochemistry by using Vectastain Universal Elite ABC Kit (Cat. No. PK-6200, Vacter Laboratories, Inc., Burlingame, California, USA) as previously described [12]. The uterus from P5.5 to P 10.5 and placenta from P11.5 to P13.5 were embedded in paraffin. Sections (5 μm) were deparaffinized in xylene and hydrated in descending grades of ethanol. TRPV6 staining involved immersion of the uterus and placenta sections in boiled citrate target retrieval buffer (0.01 M sodium citrate and 0.01 M citric acid, pH 6.0) at 100°C for 5 min. Endogenous peroxidase activity was blocked with 3% hydrogen peroxide in PBS-T (PBS containing 0.05% tween20) for 30 min. Incubation of the section in 10% normal goat serum (NGS) for 2 h at room temperature blocked nonspecific reaction. Sections were subsequently incubated with a polyclonal rabbit antibody specific to TRPV6 (#ACC-036, 0.8 mg/ml, diluted 1:100; Alomone Labs Ltd., Jerusalem, Israel) dissolved in 10% NGS, at room temperature for 2 h. A negative control was incubated with in 10% normal serum for 2 h at room temperature. After washing with PBS-T, the sections were incubated with biotinylated secondary antibody (anti-rabbit IgG; Vector Laboratories, Inc.) for 30 min at 37°C and further incubated with FAST™ 3,3'-Diaminobenzidine Tablets (#D4293, Sigma-Aldrich) for 30 min at 37°C. The sections were counterstained with hematoxylin followed by mounting with a coverslip.

### Data analysis

Data were analyzed by nonparametric one-way analysis of variance using the Kruskal-Wallis test, followed by Dunnett's test for multiple comparisons to vehicle. All statistical analyses were performed with SPSS for Windows Edition (SPSS, Chicago, Illinois, USA). *p *< 0.05 was considered statistically significant.

## Results

### Expression of TRPV6 mRNA in the uterus and placenta during pregnancy

**Figure 1:**
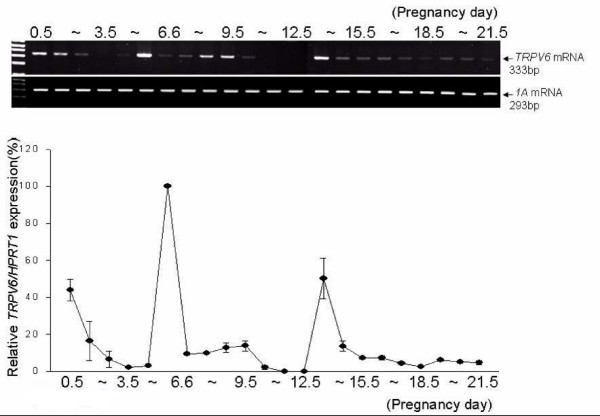
**Expression of uterine *TRPV6 *mRNA during pregnancy in rats**. Uteri of gestating rats (n = 2) were collected daily from P0.5 to P21.5, and expression of *TRPV6 *mRNA was assayed by RT-PCR (Top panel, agarose gel image) and Real-Time PCR (Bottom panel, line graph). The unattached uteri containing uterine epithelial cells were used as a RNA preparation from P1.3.5. The line graph shows the analysis of Real-Time PCR data expressed as a percentage of TRPV6/HPRT1 (mean ± SEM of duplicates).

**Figure 2:**
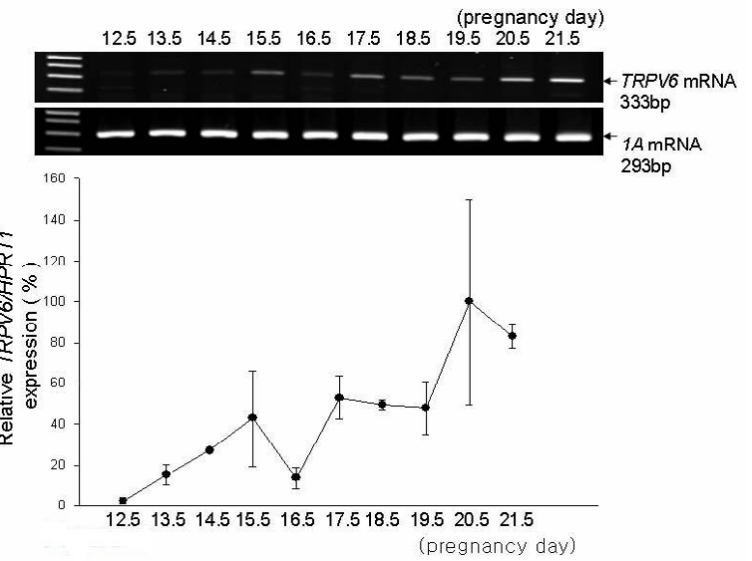
**Placental *TRPV6 *mRNA expression during pregnancy in rats**. Placentas were collected daily from P13.5 to P21.5. Placenta *TRPV6 *mRNA was examined by RT-PCR (Top panel, agarose gel image) and Real-Time PCR (Bottom panel, line graph). The line graph represents the analysis of Real-Time PCR data expressed as a percentage of TRPV6/HPRT1 (mean ± SEM of duplicates).

The expression of rat *TRPV6 *mRNA during pregnancy was examined by RT- and real-time PCR. The uteri were collected from rats at P0.5 to P21.5, and fetuses were removed. *TRPV6 *mRNA was moderately expressed at P0.5, decreased through P4.5, and then suddenly increased to the highest level at P5.5 (Figure 1). Following this at mid-gestation, *TRPV6 *expression was moderate and then increased again at P13.5. From P12.5 to P21.5, the expression of *TRPV6 *in the placenta steadily increased and expressed at the highest level at P20.5 in rats (Figure 2).

### Localization of TRPV6 protein in the uterus and placenta

**Figure 3:**
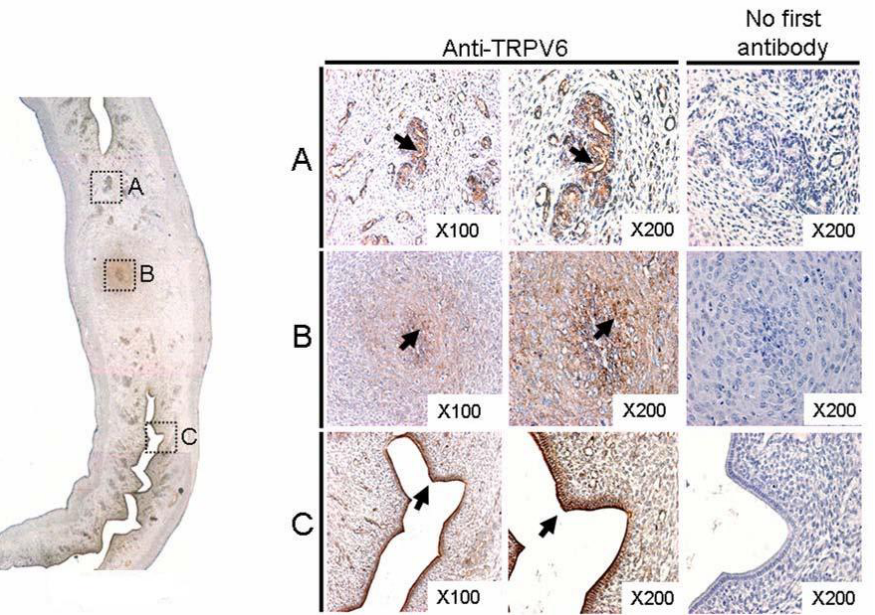
**Localization of uterine TRPV6 at P6.5**. Left panel, low power image of uterus showing representative TRPV6 immuno-positive regions restricted to three histological areas (A – C) as spotted rectangles. A, glandular epithelium; B, putative implantation site; C, epithelial layer. 'No first antibody' indicates immunoreactivity without anti-TRPV6 treatment, as a negative control. Arrows indicate immuno-positive staining.

The spatial distribution of TRPV6 protein in the rat uterus and placenta was investigated by using an anti-TRPV6 antibody, respectively, when its transcripts were shown to be expressed at relatively high levels. The rat uterus was longitudinally sectioned. TRPV6 protein was detected on the endometrial layer and glandular epithelium of the gestating uterus. TRPV6 was observed on the glandular epithelial apical layer but not on the basolateral membrane, implying that this protein may control luminal calcium ion transport (Figure 3). In addition, we observed that the site of implantation was marked by swollen uterine tissue (Figure 3). The site was strongly stained by anti-TRPV6 antibodies, supporting the notion that early embryo settlement might require the expression of TRPV6 protein (Figure 3). Between swollen areas in the rat uterus (Figure 3), TRPV6 was observed in the endometrial apical layer, consistent with our previous reports [11,12].

**Figure 4:**
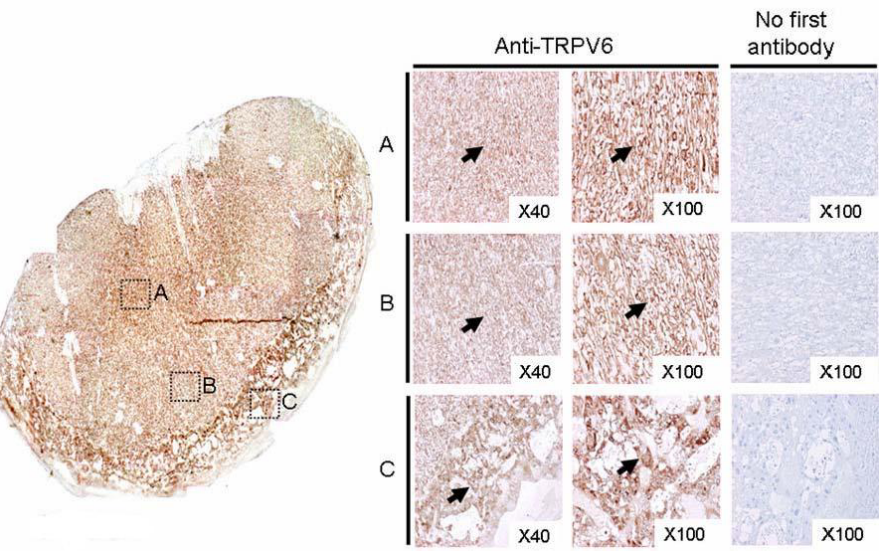
**Localization of placental TRPV6 at P20.5**. Left panel, overview of placenta divided into three distinct areas (A to C) showing TRPV6 immuno-staining as spotted rectangles. A, inner labyrinth area; B, middle labyrinth; C, spongy zone. 'No first antibody' indicates immunoreactivity without anti-TRPV6 treatment, as a negative control. Arrows indicate immuno-positive staining.

TRPV6 protein was detected throughout the rat placenta as seen in Figure 4. The placenta was separated from fetus at P20.5 and the tissue was crossly sectioned. Three areas were probed for TRPV6 expression: the inner and middle labyrinth layers, and the spongy outer layer (Figure 4A, B and 4C). Although the tissue layer on the embryo-attached-side dominantly expressed TRPV6 protein, the middle labyrinth area was also shown to express this protein. In the outer spongy layer containing giant cells, TRPV6 immuno-positive cells were also detected as demonstrated in Figure 4C.

**Figure 5:**
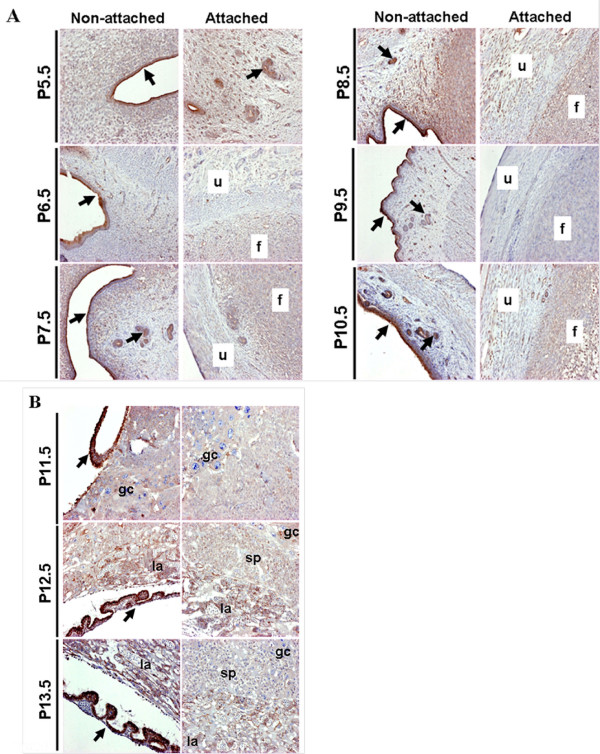
**Spatial expression of uterine (A) and placental (B) TRPV6 expression in a time-dependent manner**. A, Rat uteri from P5.5 to P10.5 were separately shown into the non-attached or attached uteri using anit-TRPV6 serum. u, attached uterus; f, placenta-like structure; arrows, epithelial and glandular cells. B, Rat placentas from P11.5 to P13.5 were presented in a time-dependent manner. la, labyrinth zone; sp, spongy zone; gc, giant cells; arrows, fetal membrane.

In a time-dependent manner, uterine TRPV6 was mainly detected in the epithelial and glandular cells of the non-attached uteri of rats (Figure 5A). The TRPV6 positive cells were observed on the attached uterus, while modest TRPV6 signal was shown in the placental-like structure regarded as the implantation site (Figure 5A). In addition, TRPV6 positive cells were broadly observed through the placenta containing the labyrinth and spongy zones in a time-dependent manner (Figure 5B). The strong signals of TRPV6 protein were detected on the fetal membranes as shown.

### Effects of sex steroid receptor antagonists on uterine and placental TRPV6 mRNA in a rat and mouse model

**Figure 6:**
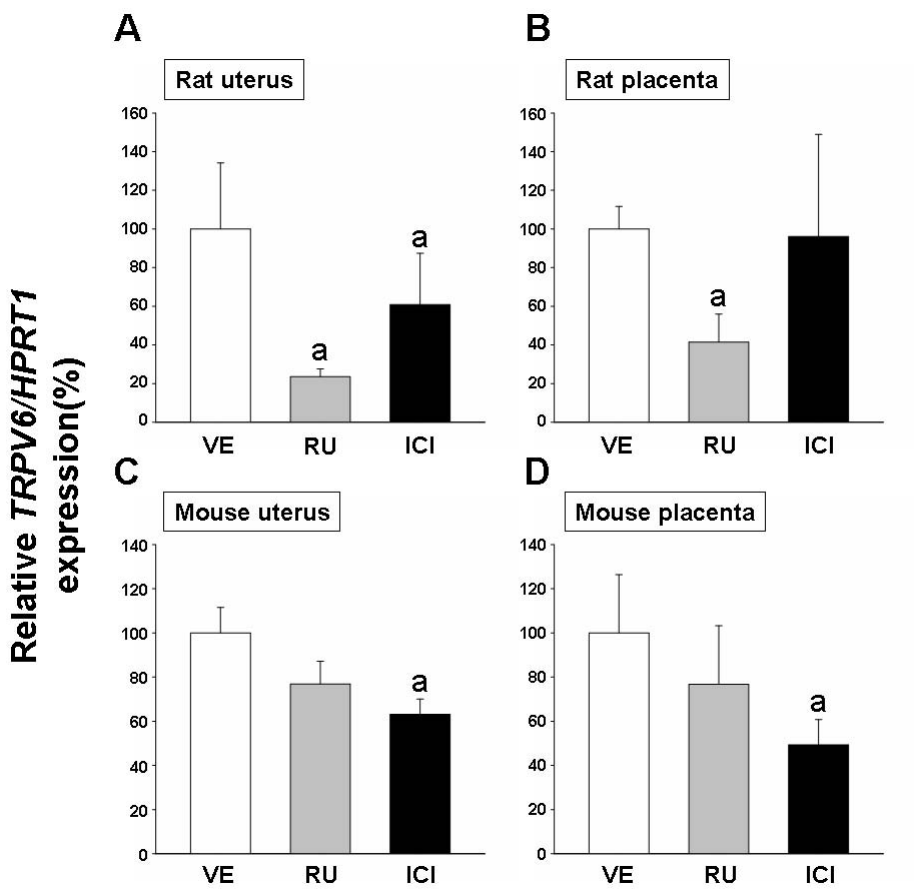
**Effects of steroid receptor antagonists on uterine and placental *TRPV6 *mRNA expressions**. Panel A (uteri at P5.5) and B (placenta at P20.5) presented the rat *TRPV6 *mRNA levels. Four groups of pregnant rats (n = 4 per group) were treated with ethanol as a negative control (VE), progesterone receptor antagonist (RU, 2.5 mg per rat), or estrogen receptor antagonist (ICI, 0.5 mg per mouse). Panel **C **(uteri at P10.5) and D (placenta at P10.5) showed the mouse *TRPV6 *mRNA expressions. Four groups of pregnant mice (n = 4 per group) were treated with ethanol as a negative control (VE), progesterone receptor antagonist (RU, 25 μg per mouse) or estrogen receptor antagonist (ICI, 2 μg per mouse). Murine *TRPV6 *mRNA levels were examined by real-time PCR. The bar graph represents the analysis of real-time PCR data expressed as a percentage of TRPV6/HPRT1 (mean ± SEM of duplicates). a, statistically significant compared to a vehicle (*P *< 0.05).

Recently, uterine *TRPV6 *was shown to be regulated by progesterone in immature and non-pregnant rats [11], whereas its expression in a mouse model was shown to be controlled by estrogen [12]. To clarify which hormone mediates uterine *TRPV6 *expression, the rats and mice were treated with a single treatment of RU or ICI to invoke progesterone and estrogen receptor antagonism conditions, respectively. In the uterus at P5.5 (maximum expression levels), RU treatment resulted in a significant inhibition of *TRPV6 *mRNA expression in the rat uterus. In addition, ICI significantly reduced *TRPV6 *transcription in this tissue (Figure 6A). In the rat placenta, hormonal regulation was tested at P19.5 after a single treatment with RU or ICI. Treatment with RU significantly down-regulated the expression of placental *TRPV6 *at P20.5, while ICI did not alter its expression in the rat placenta (Figure 6B), suggesting that placental *TRPV6 *transcription in gestating rats may be controlled by progesterone and its receptors.

Previously, we reported that uterine *TRPV6 *transcription in mice was regulated by estrogen via estrogen receptor α [12], but its placental regulation during pregnancy has not been investigated. In the previous study, steroid hormone-induced regulation of murine *TRPV6 *transcription was shown by using ICI or RU at P10, a point in gestation when *TRPV6 *was previously reported to be highly expressed [12]. As shown in Fig 6C, treatment with ICI significantly reduced uterine *TRPV6 *expression, whereas RU did not affect uterine *TRPV6 *transcription levels in the mouse uterus. In addition, *TRPV6 *gene was significantly down-regulated by ICI, while RU did not alter its mRNA levels in the mouse placenta (Figure 6D). Taken together, these data indicate that uterine and placental *TRPV6 *expression may be primarily regulated by estrogen during non-pregnancy and pregnancy in mice. This regulation of *TRPV6 *expression by estrogen in mice is in contrast to progesterone-mediated regulation in rats.

## Discussion

Several uterine calcium-regulating genes are alternatively expressed during estrous cycle and pregnancy [23]. In the implantation period, calcium-binding proteins appear to play a central role [8,12,24-33]. Calbindins are required during the early phase of embryo implantation, implying that the regulation of calcium availability in the vicinity of the implanting embryo is critical for successful implantation [8,9]. Although the importance of calbindins in implantation has been established, the precise role of calcium ions during implantation remains unclear. Other calcium processing genes have recently been identified in female reproductive organs, and TRPV6 is an interesting protein in elucidating uterine process of calcium [11,12,34].

Uterine expression of *TRPV6 *varies during the estrous cycle in rodents. For instance, rat *TRPV6 *transcripts in the uterus were highly expressed at diestrus [11], while mouse *TRPV6 *mRNA was highly expressed at estrus [12]. Distinct their expression patterns indicate that uterine *TRPV6 *is differentially regulated during the estrous cycle in rats and mice. During pregnancy, mouse *TRPV6 *mRNA is actively expressed with maximal expression observed in the middle of gestation, followed by a reduction in the late period. Immediately prior to birth, another high level of *TRPV6 *expression is observed, which disappears during lactation [12]. In the present study, rat uterine *TRPV6 *was modestly expressed at P0.5, which corroborates previous reports; and this gene was up-regulated by progesterone at the diestrous stage in a rat model [11]. In days after P0.5, *TRPV6 *expression gradually decreased and was undetected at P4.5. However, at P5.5 a substantial increase and peak in *TRPV6 *expression was observed. This time point coincides with the earliest sign of the implantation attachment reaction which occurs around P4 or P5 in rodents [35]. Therefore, the maximal expression of *TRPV6 *gene at P5.5 may be related to the regulation of uterine calcium ion concentration required for successful implantation. Calbindin-D9k, another uterine calcium associated protein, gradually increases at pregnancy day 16.5, peaks at day 18.5, and declines at birth and the beginning of lactation [17]. A comparison of uterine *TRPV6 *and *calbindin-D9k *time-dependent expressions in pregnant rats suggests that these proteins may share in the function for maintaining uterine calcium concentrations during pregnancy.

In mice, uterine calbindin-D9k showed a gradual increase in mRNA expression during late pregnancy (from day 12.5 to 18.5), followed by a decline at birth and during lactation [17]. It has also been reported that serum estrogen levels increase at the end of pregnancy and decline sharply following birth and during lactation, whereas serum progesterone levels remain constant throughout late gestation and lactation [17]. With regard to these studies, it is possible that increased serum estrogen levels induce uterine *TRPV6 *transcription at birth and during pregnancy, and that TRPV6 ceases during lactation when secreted estrogen is no longer present. It was previously shown that mouse *TRPV6 *expression in the placenta initially follows the pattern of uterine expression, with an induction in the middle of gestation (P10.5 and P14.5) but not at the end of gestation [12]. *TRPV6 *seems to have a positive influence on transplacental calcium ion concentrations and to be a crucial factor in fetal development, because it is highly expressed in human placenta as compared to levels in other organs [36]. In the present study, placental expression of *TRPV6 *gene steadily increased from P12.5 to P20.5 before labor in rats. Taken together, these results imply that constitutive expressions of TRPV6 and/or other calcium processing genes may play a crucial role in transporting calcium ions from maternal to fetal compartment during pregnancy. In addition, the changes in the uterine transcript levels of TRPV6 as a function of pregnancy may be largely a reflection of myometrial changes in its expression in both rats and mice.

Rat TRPV6 protein was detected in the endometrial layer, glandular epithelium and implantation site of the gestating uterus. This spatial pattern of TRPV6 expression between uterus and placenta was previously demonstrated [12]. In parallel with TRPV6, rodent calbindin-D9k was detected predominantly in luminal epithelial cells of the uterus of pregnant rats [17,37]. The similar spatial distribution of these gene products further supports the concept of a complementary function for TRPV6 and calbindin-D9k in the control of calcium ions during pregnancy. In rodent models, placental localization of TRPV6 protein in the labyrinth and spongy zone implies that this protein may take part in transporting calcium ions between fetus and dam. In regard to its expression levels during pregnancy, the expression of TRPV6 protein would be highest in the myometrium as shown in this study.

Progesterone has been implicated as a major regulator in the expression of *TRPV6 *during the rat estrous cycle [11]. The progesterone receptor antagonist (RU) significantly blocked progesterone-induced *TRPV6 *gene transcription [11]. The expression of uterine *TRPV6 *mRNA at P5.5 was completely inhibited by RU treatment, and partially inhibited by treatments with ICI. Partial inhibition of *TRPV6 *expression by ICI may be attributed to the action of estrogen receptor at estrogen response elements (EREs) found in the promoter region of the progesterone receptor gene. Although ICI partially down-regulated *TRPV6 *gene in the rat uterus, progesterone and its receptor appear to primarily control uterine *TRPV6 *transcripts in this tissue during pregnancy.

A recent study provides the first in vivo evidence that TRPV6 is involved in maternal-fetal Ca^2+ ^transport, proposing that TRPV6 functions as a Ca^2+ ^entry pathway, which is critical for fetal Ca^2+ ^homeostasis [16]. In addition, a phenotype of mice with targeted disruption of *TRPV6 *gene was reported. *TRPV6 *knockout (KO) mice showed disordered Ca^2+ ^homeostasis and reduced fertility, and deficient maternal-fetal transfer of calcium ions caused abnormal calcium ion deficiency during embryonic development [38]. In this study, placental TRPV6 expression is expected to enhance the rate of maternal transfer of calcium ions to the fetus during pregnancy [38]. In the present study, the expression of placental *TRPV6 *mRNA in rats was blocked by RU treatment, but no significant inhibitory effect was observed for ICI treatments, suggesting that placental *TRPV6 *expression is under the sole control of progesterone via a progesterone receptor-mediated pathway. However we cannot rule out the possibility that the difference in their doses may account for the observed difference between rats and mice.

In contrast to rats, it was previously reported that murine uterine *TRPV6 *transcription at normal estrous cycles was totally dependent on estrogen [12]. Uterine *TRPV6 *mRNA levels increased at mid and late pregnancy, and were strongly induced at mid-pregnancy in the labyrinth and spongy zones of the placenta, and in the fetal membrane [12]. To examine an expression pattern of TRPV6 transcripts by steroids compared to its level in rats during pregnancy, we treated the mice with steroids, E2 and P4, and isolated the identical tissues from them to measure TRPV6 levels. In the present study, sex steroids controlled uterine and placental *TRPV6 *expression during pregnancy. The expression of uterine *TRPV6 *mRNA at P10 was blocked by ICI, and placental *TRPV6 *transcription was blocked by ICI, suggesting that the expression of *TRPV6 *was primarily regulated by estrogen in gestating uteri and placenta during pregnancy in mice. These results suggest that *TRPV6 *may be distinctly regulated by different sex steroids, progesterone in rats and estrogen in mice, in spite of shared similarities in their chromosomal make-up [12,20,39].

These results show that uterine and placental *TRPV6 *mRNA levels in rats appear to cyclically change during pregnancy, suggesting that TRPV6 protein may participate in the implantation process. It was of interest to note that uterine *TRPV6 *mRNA is only expressed in placenta-unattached areas of the uterus, indicating that *TRPV6 *has at least two functions: implantation of the embryo and maintenance of pregnancy. In rats, *TRPV6 *transcription appeared to be solely controlled by P4 through PRs, while its level was regulated by E2 via ERs in mice. In both rodents, uterine TRPV6 immunoreactivity was observed in luminal and glandular epithelial cells, and in the placenta TRPV6 was detected in the labyrinth and spongy zone. The juxtaposition of uterine and placental TRPV6 expressed in these tissues can support a novel concept that TRPV6 participates in transporting calcium ions between the maternal and fetal compartments. In conclusion, TRPV6 gene may function as a key element in controlling calcium transport in the uterus between the embryo and the placenta during pregnancy.

## Competing interests

The authors declare that they have no competing interests.

## Authors' contributions

BL carried out the overall experiments including animal treatments and molecular experiments. GL carried out the animal treatments and molecular experiments with BL, and drafted the manuscript. EJ participated in the real-time PCR and immunohistochemical analysis. KC participated in the design of the study, performed the statistical analysis and helped to finalize the manuscript. EJ* designed and coordinated the overall study as a corresponding author and helped to draft the manuscript. All authors read and approved the final manuscript.
